# Pulmonary vein isolation treats symptomatic AF in a patient with Lamin A/C mutation: case report and review of the literature

**DOI:** 10.1007/s00392-020-01616-x

**Published:** 2020-03-06

**Authors:** Ann-Kathrin Rahm, Patrick Lugenbiel, Marco Ochs, Benjamin Meder, Dierk Thomas, Hugo A. Katus, Eberhard Scholz

**Affiliations:** 1 Department of Cardiology, Medical University Hospital Heidelberg, University of Heidelberg, Im Neuenheimer Feld 410, 69120 Heidelberg, Germany; 2 Heidelberg Center for Heart Rhythm Disorders, Heidelberg, Germany; 3 DZHK (German Centre for Cardiovascular Research), Partner Site Heidelberg/Mannheim, Heidelberg, Germany

**Keywords:** Laminopathy, *LMNA*, Lamin a/c, Ablation, Pulmonary vein isolation, Atrial fibrillation

## Abstract

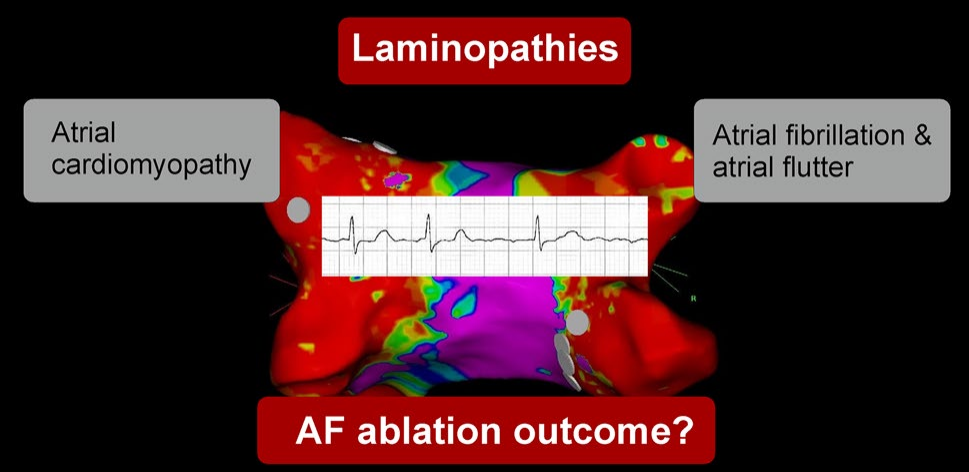

Sirs:

Lamins are the nuclear intermediary filaments that stabilize the nucleus in eukaryotic cells. They are encoded by the gene *LMNA* giving rise for lamin A and C by a splice variant. Mutations lead to several diseases from progeria to skeletal muscular dystrophy. In the heart, mutations in the *LMNA* gene may cause cardiomyopathy, conduction disease, atrial and ventricular arrhythmias as well as sudden cardiac death (SCD) [1]. Patients with laminopathies often present with a phenotype of dilated cardiomyopathy [2] and as in most other cardiomyopathyies the incidence of atrial fibrillation (AF) is increased in this patient collective [3]. Little is known so far about the effect of pulmonary vein isolation (PVI) and its outcomes as a symptomatic treatment for atrial fibrillation (AF) in this patient cohort with *LMNA* mutations at an early stage of atrial remodeling [4].

In general, Laminopathy in the heart is associated with a poor prognosis, related to heart failure due to dilated cardiomyopathy and sudden cardiac death, caused by ventricular arrhythmia or conduction block. First phenotypic changes include conduction abnormalities like AV block type I or supraventricular arrhythmias [5, 6]. Bradycardic and tachycardic supraventricular arrhythmias including atrial fibrillation (AF) often precede decades before the development of heart failure due to dilated cardiomyopathy [7]. In contrast to general population, where AF shows an increasing incidence in the elderly, in laminopathy patients AF occurs early at an age even below 30 years and AF in the young should raise the suspicion of an underlying lamin mutation. AF is often the first cardiac manifestation of the laminopathy, as in our presented patient below. In laminopathy AF is found in around 60% of patients increasing to around 90% in patients who progressed to manifest dilated cardiomyopathy [8]. On the cellular level various factors are known for promoting fibrosis [8, 9]. In laminopathies mutations in lamin A/C lead to structural remodeling and fibrosis of the conduction system and the myocardium. In the atria ongoing remodeling processes will promote arrhythmia and finally may also lead to atrial paralysis when functional atrial myocardium has been completely replaced by fibrous and adipose tissue [10]. Ventricular arrhythmias can occur at any stage of the disease. The phenotype within a given family may vary in cardiac expression as well as chronology of signs and symptoms.

Here, we report on a 37-year-old female patient presented at our center in 2017 for family screening of numerous cases of SCD. On presentation, she was asymptomatic and her 12-lead resting ECG revealed an atrioventricular block (AVB) grade I and low atrial amplitude suggestive for a laminopathy (Fig. 2a). Echocardiography displayed a normal left ventricular ejection fraction and cardiac MR confirmed normal function, but revealed septal intramural late gadolinium enhancement (LGE) typical for laminopathy (Fig. 1) [11]. Also in both atria a LGE signal could be observed, showing already developed fibrosis and matching the changes in 12-lead ECG. The patient’s genetic testing showed a lamin A/C nonsense mutation. Consecutively, because of an already existing AVB I° and sinusbradycardia, a 2-lead ICD was implanted for primary prophylactic reasons in this patient with positive family history for SCD [12].

**Fig. 1:**
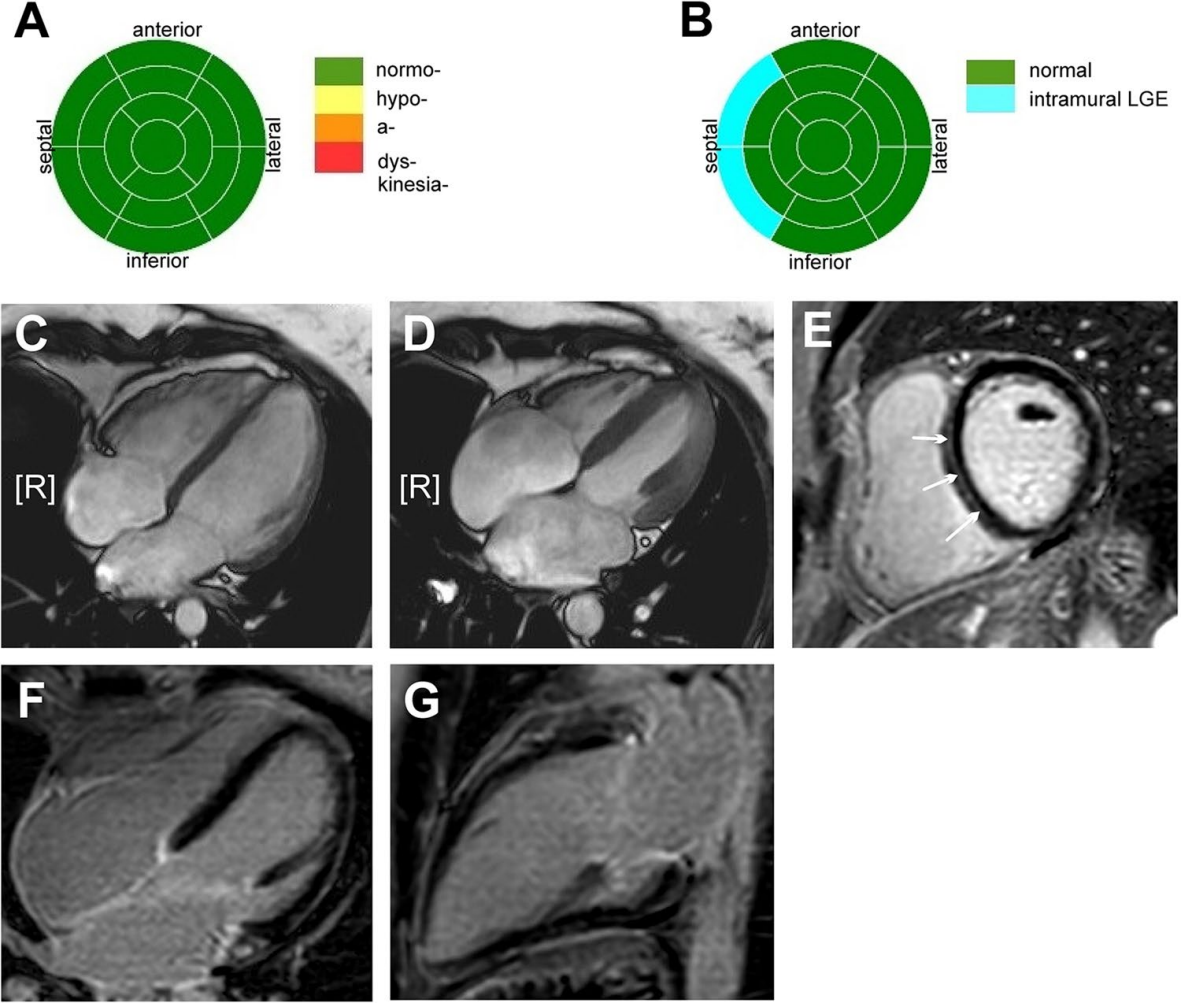
**a**–**e** The cardiac MRI prior to 2-lead ICD implantation: **a** bulls eye blot with normal wall movements with a normal left ventricular ejection, **b** bulls eye blot of typical LGE pattern in the intramural septum, **c** enddiastolic and **d** endsystolic four chamber view and **e** short axis view of typical midwall fibrosis with LGE (marked with white arrows). **f** LGE in four chamber view, **g** LGE in two chamber view: Also in the atria (LA > RA) an LGE signal typical for atrial fibrosis can be detected

One year later in the beginning of 2018, symptomatic episodes of AF (EHRA III) were documented in the holter of the ICD (AHRE) [13] and in 12-lead ECGs (Fig. 2b). Initially betablockers were prescribed for rate control, but were not well tolerated due to hypotension. Treatment options were intensively discussed with the patient and she was informed that little evidence regarding the outcome of PVI in patients with laminopathy is existent. The latest ESC and HRS guidelines on AF do not comment on treatment of this specific patient collective [14, 15] and by an intensive literature review only one case of successful radiofrequency (RF) ablation in a patient with laminopathy was found [16]. Despite low evidence, due to severe symptomatic AF episodes and only mild atrial dilatation (42 mm diameter in TTE), the individual treatment decision was made: In April 2018 the patient received cryoballoon PVI using the second generation cryoballoon (Medtronic, Dublin, Ireland) in typical anatomical configuration with two left- and two right-sided pulmonary veins and standard setting with an esophageal thermo probe (Fig. 3). Successful entrance and exitblock of the veins were achieved. Retrospectively, the holter in the ICD depicted a typical heart rate increase after PVI (Fig. 5, heart rate). In the post-PVI blanking period symptomatic AF episodes still occurred with a decreased frequency after 1 month, but symptoms did not completely subside. In July 2018, a Re-PVI was performed using CARTO 3 Mapping system (CARTO3™, Biosense Webster, Diamond Bar, CA, USA). Electroanatomical mapping revealed a re-connection of the LSPV and RIPV after PVI and displayed a not severely remodeled left atrium with most areas besides the isolation lines above voltages of 0,5 mV. Both veins were successfully re-isolated (Fig. 4), the LSPV in the superior and RIPV in the posterior-inferior area with documented exit and entrance block. Monitoring of the daily heart rates after the Re-PVI showed a discontinuation of nightly heart rate spikes (Fig. 5, heart rate), suggestive of trigger PV activity before. After additional initiation of low-dose betablockade under protection of the implanted two-lead ICD (Medtronic, Dublin, Ireland), symptomatic AF episodes were abolished (Fig. 5) and a drop in heart variability can be observed.

**Fig. 2:**
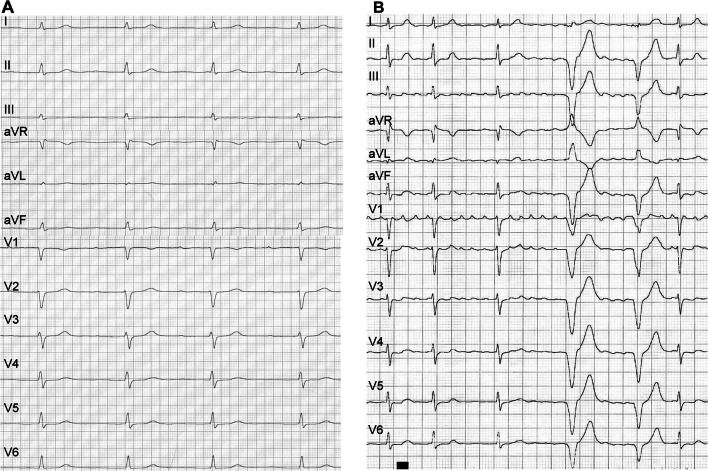
**a** The patient’s baseline ECG with AV block type I and **b** AF with intermittent ventricular stimulation of the 2-lead ICD

**Fig. 3:**
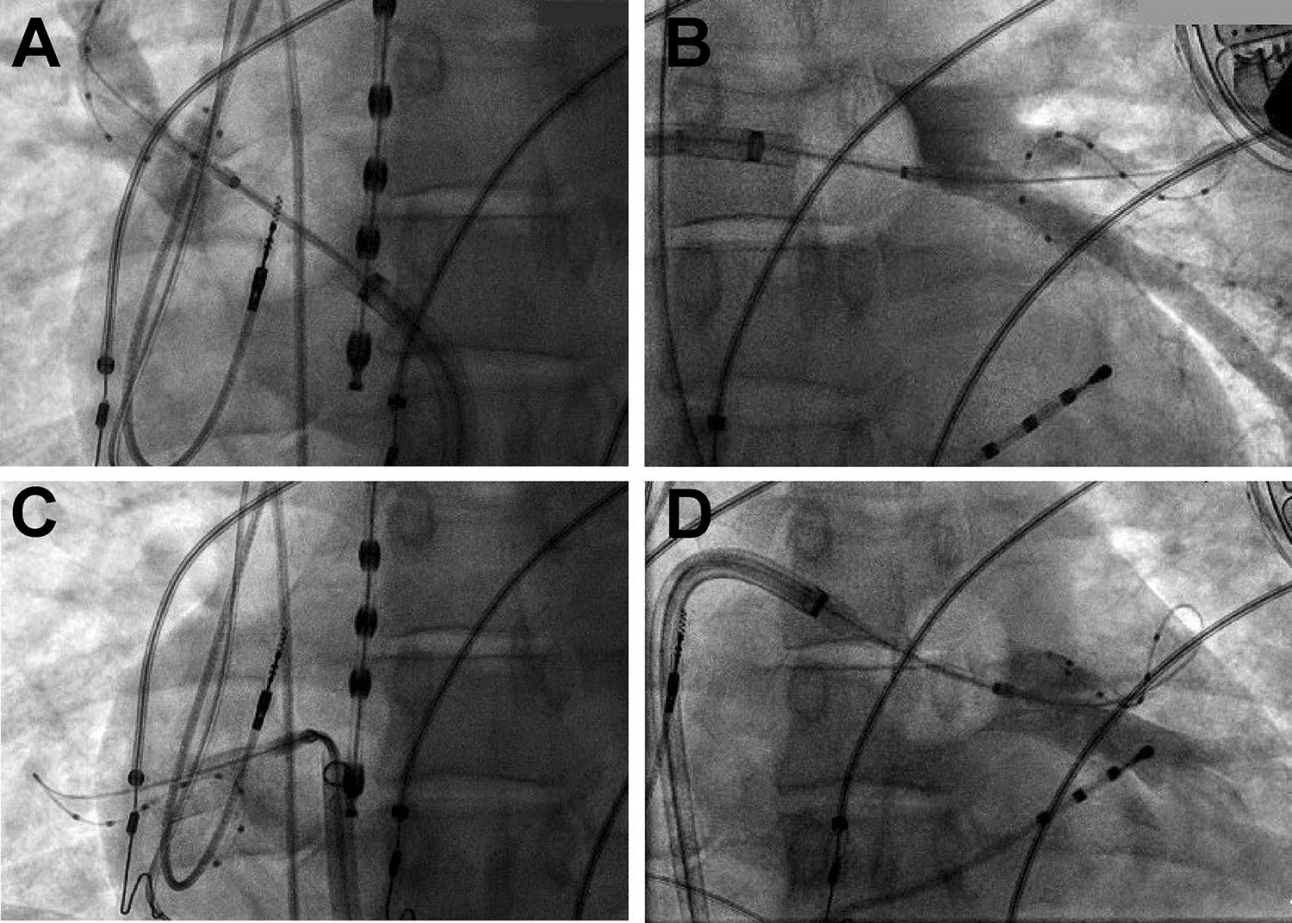
PV angiography and documentation of cryoballoon occlusion in projection **a** RSPV, **b** LSPV, **c**, RIPV, **d** LIPV with more distal mapping catheters for improved stabilization. Isolation was confirmed also by positioning the lasso in the pulmonary vein ostia. Temperature probe in the esophagus, quadripolar CS-electrode and two-lead-ICD electrodes are also visible

**Fig.4:**
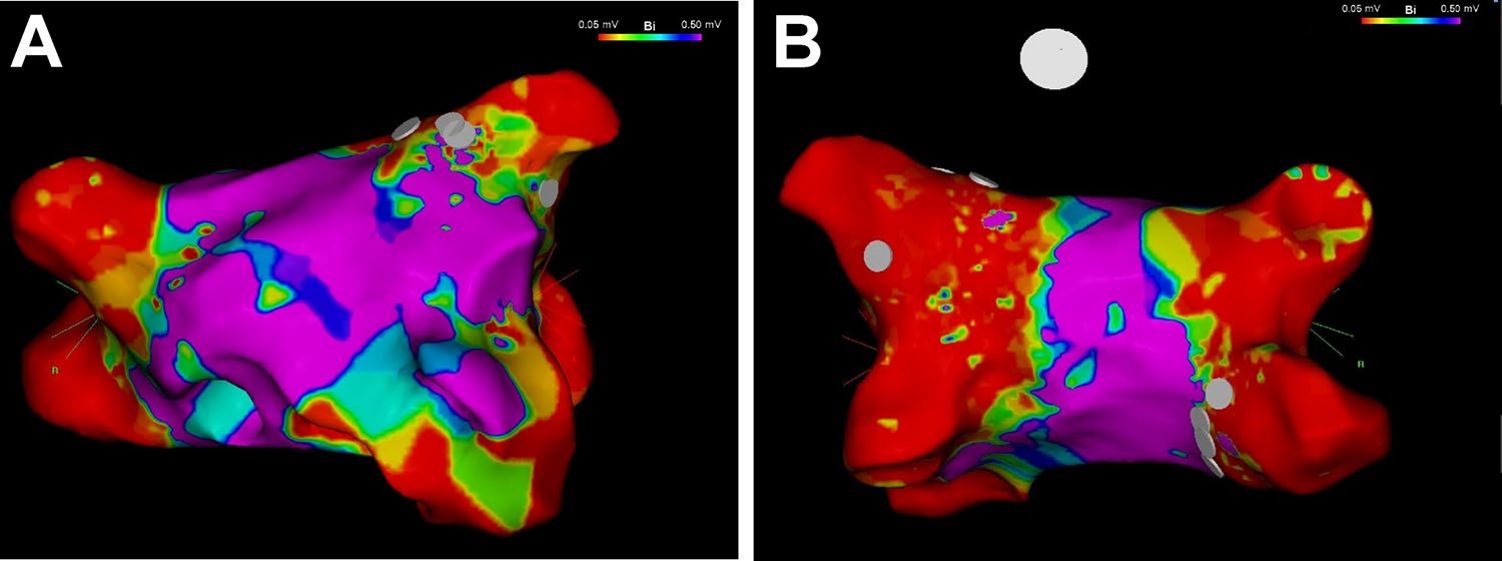
Electroanatomical mapping for Re-PVI using CARTO 3 (CARTO3™, Biosense Webster, Diamond Bar, CA, USA). Maps show standard bipolar configuration, marked in purple voltage above 0.5 mV, marked in red voltage below 0,05 mV defining scar region. **a** Map from anterior–superior view, **b** Map from posterior view. Marked in grey are the ablation sites on the LSPV and RIPV

**Fig. 5:**
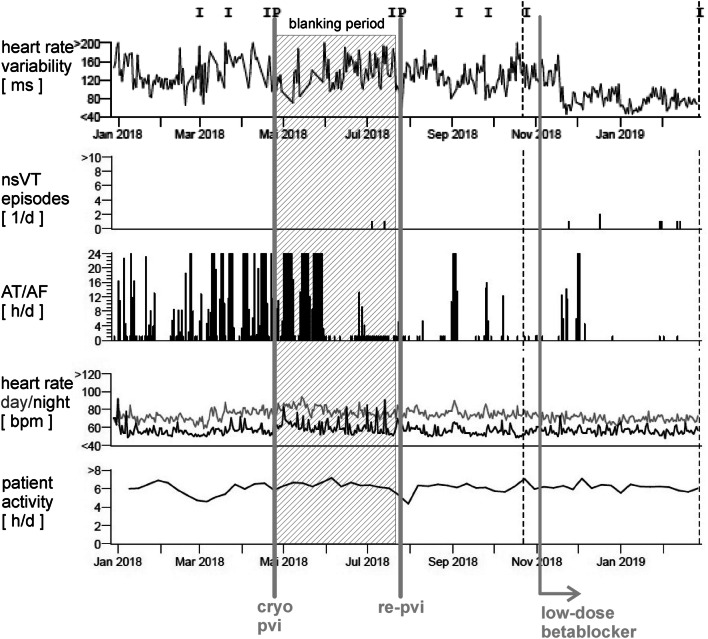
Cardiac monitor of the 2-lead ICD (Medtronic, Dublin, Ireland). Marked with “P” and grey lines is the programming of the ICD to deactivated antitachycardia therapy within the ablation procedures. AT/AF episode recording shows a significant decrease in AF burden after the PVI procedures compared to the beginning of 2018

We report a case of successful treatment of symptomatic AF with a two-step approach of PVI in a patient with lamin A/C mutation. In this young patient treatment with PVI did not only reduce AF episodes but most importantly improved quality of life during the short-term follow-up. Despite the fact that mid- to long-term effects of PVI is still missing in our patient, it is nevertheless important to communicate the need for further case studies and randomized-trials in specialized centers to assess the time course of atrial remodeling and the long-term effects of AF ablation in this highly arrhythmogenic patient collective. Numerous patients have undergone PVI procedures as in recent ablation registries and studies [17–29] and it can be assumed that laminopathy patients have been included without knowing or including their genetic background. It may be worthwhile to re-analyze existing data of ablation registries regarding the existence of laminopathies. Reduced symptoms and AF itself caused remodeling a decade before worsening of left ventricular function may prove to be beneficial in the end.
